# A Review on the Inertial Measurement Unit Array of Microelectromechanical Systems

**DOI:** 10.3390/s24227140

**Published:** 2024-11-06

**Authors:** Jiawei Xuan, Ting Zhu, Gao Peng, Fayou Sun, Dawei Dong

**Affiliations:** School of Automation, Guangxi University of Science and Technology, Liuzhou 545006, China; 20230201015@stdmail.gxust.edu.cn (J.X.); 221077077@stdmail.gxust.edu.cn (G.P.); 20230203013@stdmail.gxust.edu.cn (F.S.); 20230202007@stdmail.gxust.edu.cn (D.D.)

**Keywords:** microelectromechanical systems, inertial measurement unit array, data fusion

## Abstract

In recent years, microelectromechanical systems (MEMS) technology has developed rapidly, and low precision inertial devices have achieved small volume, light weight, and mass production. Under this background, array technology has emerged to achieve high precision inertial measurement under the premise of low cost. This paper reviews the development of MEMS inertial measurement unit (IMU) array technology. First, the different types of common inertial measurement unit arrays are introduced and the basic principles are explained. Secondly, IMU array’s development status is summarized by analyzing the research results over the years. Then, the key technologies and corresponding development status of IMU array are described, respectively, including error analysis modeling and calibration, data fusion technology, fault detection, and isolation technology. Finally, the characteristics and shortcomings of the past research results are summarized, the future research direction is discussed, and some thoughts are put forward to further improve the accuracy of the IMU array.

## 1. Introduction

Microelectromechanical electro mechanical systems (MEMS) inertial devices have many advantages such as low cost, small size, light weight, low power consumption, and easy large-scale production [1], and have been widely used in the field of inertial navigation. However, the measurement accuracy and reliability are low, which cannot be ignored. Therefore, it is an important research direction to improve the accuracy of MEMS inertial devices while making full use of their advantages [2].

The use of low cost, low precision and low reliability MEMS inertial devices to manufacture high-precision and high-reliability products, and then achieve high-precision and high-reliability navigation goals, has become a hot topic in the field of inertial navigation. There are two main ways to achieve the above goals, one is to improve the accuracy of a single MEMS inertial device, and the other is to use multiple low-precision MEMS inertial devices to improve the accuracy. As early as 2003, David S. Bayard and Scott R. Ploen in the United States proposed a method for fusing multiple gyroscopes to significantly improve performance [3,4]. Under the influence of this idea, later researchers in the field of inertial navigation further proposed MEMS virtual gyroscope array [5], accelerometer array, inertial sensor array [6], IMU array [7], etc., all of which are based on Newton’s law of inertia. It has become an important research direction in the field of inertial navigation to construct inertial measurement unit array using multiple MEMS inertial devices, and then reduce error, improve accuracy and increase reliability through data fusion technology.

## 2. Principle and Research Status

The inertial measurement unit technology originated from David S. Bayard and colleagues at NASA’s Jet Propulsion Laboratory in 2003, with the concept of “virtual gyroscopes”. They theoretically proposed a method to enhance accuracy for MEMS gyroscopes by processing outputs from multiple gyroscopes. Through computer simulations, the fused gyroscope accuracy was improved by a factor of 173. They further demonstrated that when N gyroscopes are combined into an array, the drift of the combined gyroscope array can be significantly reduced, thus enhancing performance [3,4].

**Figure 1:**
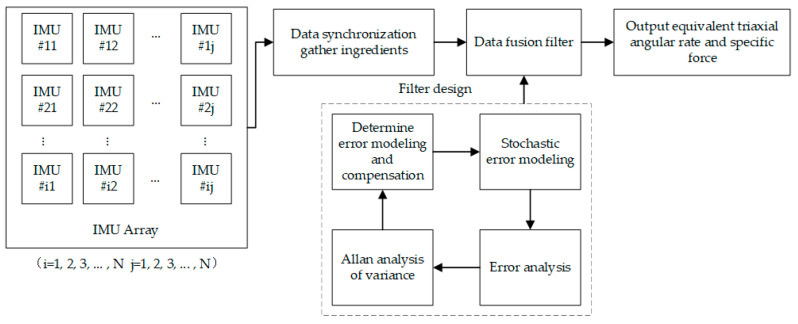
MEMS IMU array technology schematic.

IMU array technology refers to a technical solution that adopts multiple inertial measurement units to form an array, measure the same acceleration and angular velocity signals, analyze and model the signals collected by each IMU, and use data fusion technology to fuse the measurement data of multiple inertial sensors into the output of the IMU array, thereby improving the accuracy, as shown in Figure 1.

The inertial measurement unit adopts the principle of Newton’s law of inertia, which is used to detect the acceleration, angular velocity, and other information of the measured object, and convert the collected physical signal into electrical signal output. Inertial sensors include accelerometers and gyroscopes, which measure acceleration and angular velocity information, respectively. As the core part of the inertial system, the inertial sensor is an important factor affecting the whole inertial system. According to Newton’s law of inertia, velocity and position information can be obtained by integrating acceleration information and double integration, and angular information can be obtained by integrating diagonal velocity information. However, the main problem is that errors grow rapidly with the double integration, so that the system is not reliable [8], which seriously affects the navigation accuracy. Therefore, by integrating the above information and combining the positioning and orientation navigation algorithms [9], the position, speed, attitude, and other information of the measured object can be obtained.

In 2004, Lam et al., based on dynamic modeling of random noise of MEMS inertial devices, designed a dual-path compensation method that includes reference external correction and dynamic detection to identify noise parameters in real-time, which is used to provide filtering and noise elimination, effectively eliminate the noise source of MEMS sensors, and improve the performance. This method also provides a new idea for the internal self-calibration design of MEMS sensors [10].

In 2006, Min Hu from Northwestern Polytechnical University proposed a MEMS virtual gyroscope system based on array technology in his Master’s thesis, the virtual gyroscope based on the data fusion of multiple similar sensors. He designed a micro-gyroscope with high quality factors under normal pressure in hardware, and then compensated it in software. Three such gyroscopes are formed into a gyroscope array by Kalman filter and then fused into a high-precision virtual gyroscope [11]. Since then, Beijing University of Aeronautics and Astronautics, Beijing Institute of Technology, Wuhan University, and other institutions have also carried out a series of studies on MEMS IMU array technology.

In 2008, Peter A. Stubberud of the University of Nevada, United States, proposed A new extended Kalman filter algorithm to solve the dimensional disaster of nonlinear fusion of multiple MEMS inertial devices. The dynamic information of each sensor is fully utilized to effectively improve the fusion accuracy [12].

With the further development of research, array technology has been applied in engineering practice. Since 2008, American companies Tanenhaus and Associates have successively launched a number of low-cost and high-precision inertial navigation systems based on array technology, which are used in intelligent munitions, micro-missiles, micro-UAVs, and other applications [13].

**Figure 2:**
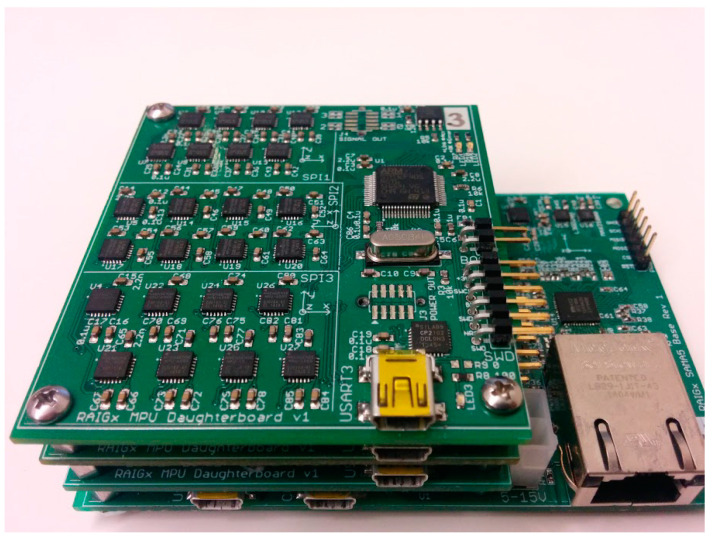
The 72 MEMS gyroscope arrays [14].

In 2015, John Wang et al. from the University of Michigan in the United States integrated 72 MEMS gyroscopes on a three-layer development board. As shown in Figure 2, a hidden Markov model was adopted to model and fuse the gyroscope array. Experiments showed that the gyroscope accuracy was improved by 50% compared with Kalman filter, which significantly improved its performance [14].

In 2018, Owais Talaat Waheed et al. from Khalifa University in the United Arab Emirates designed a vector processor based on Artix-7 FPGA, which solved the problem of scalability of IMU array sensor fusion and realized real-time and high-throughput IMU sensor array fusion [15]. The following year, they further proposed domain-specific architecture for IMU array data fusion, using Kalman filters to combine and predict the output and internal states of IMU arrays, and designed a compact data fusion processing system based on a combination of model approximation techniques and domain-specific processors. Compared with general-purpose processors, it greatly improves the efficiency of data fusion [16]. It marks that the research of MEMS IMU array has entered the application stage.

In 2019, Chen et al. from Shanghai Micro-Satellite Engineering Center proposed an information fusion method for a micro-nano satellite gyroscope array system, which used time series modeling and Kalman filtering to filter the error signal of gyroscope and reduce the error of MEMS gyroscope. The fused gyroscope array met the application requirements of micro-nano satellite [17]. The application of MEMS gyroscope array in micro-nano satellite is expanded.

In 2020, Jing of Soochow University conducted a study on a pedestrian navigation system based on array inertial sensor in their Master’s thesis. They built a gyroscope array containing 32 MPU9250 in hardware, designed a pedestrian navigation system algorithm in software, corrected the gyroscope error through complementary filtering, and then carried out inertial navigation solution. Finally, Kalman filter algorithm based on zero speed interval was used for error correction, so as to realize pedestrian navigation function [18], which expands the application of IMU array in the field of wearable devices.

**Figure 3:**
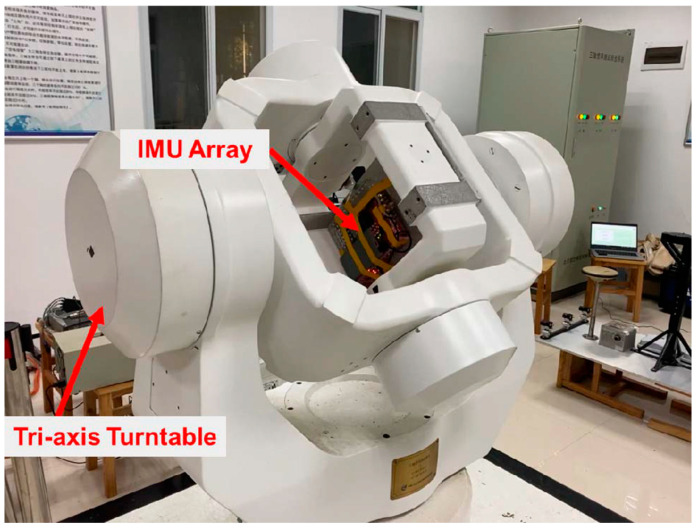
The IMU array was calibrated by a high-precision three-axis rotary table in Wuhan University [19].

In 2021, Professor Niu’s team at Wuhan University used a high-precision three-axis rotary table to calibrate the constant error of MEMS IMU array. As shown in Figure 3, the navigation accuracy of IMU array after error compensation was increased by 3.4 times, close to the theoretical limit value of 4 times. In the sports car experiment, the average position error of pure inertial navigation with GNSS interrupted for 30 s was only 1.85 m [19]. It provides experimental support for the application of IMU array in the inertial navigation field.

In 2022, Liang Siyuan et al. from Xi’an University of Posts and Telecommunications realized IMU array calibration without external calibration devices by manually adjusting IMU array attitude, and the measurement accuracy of the array after error compensation was improved by about 10 dB [20], providing a new research idea for the IMU array self-calibration method.

In 2023, Yang et al. from Wuhan Institute of Technology integrated the Global Positioning System (GPS) and IMU sensors, built a Kalman filter platform using FPGA, and built a loosely coupled integrated navigation system, which is more accurate than single sensor positioning [21]. The application of IMU array in integrated navigation is greatly promoted.

In 2024, Cao et al. from Sichuan University of Light Chemical Technology used a sliding variance detector to divide the angular velocity into a static interval and a moving interval, improved the traditional polyhedral static calibration method, calibrated each IMU separately, and then compensated the velocity residual of the zero-velocity interval after the zero-velocity update to improve the average noise performance of the IMU array [22]. They applied this method to the error compensation of IMU arrays in pedestrian navigation systems, which improved the accuracy of pedestrian navigation and further promoted the development of IMU arrays in the field of wearable devices.

Through the above analysis of the research status of MEMS IMU array technology, it can be seen that the research mainly focuses on the following aspects: (1) in the calibration method, most researchers use a high-precision turntable to calibrate the array, and some of them put forward the self-calibration method of the array; (2) in terms of data fusion technology, research methods focus on the Kalman filter method and its extension, but some researchers have begun to use neural networks for data fusion of arrays; (3) in practical applications, including applications in integrated navigation, satellites and wearable devices.

Although some research work has been carried out in MEMS inertial measurement unit array technology, there are still some problems to be solved. First, the process of using a high-precision rotary table to calibrate the array is complicated and expensive, so it is necessary to further study the new method of calibrating the array to reduce the cost of calibration. Secondly, the existing data fusion methods have their own advantages and disadvantages. How to further improve the fusion accuracy and reliability of MEMS IMU arrays needs to be optimized and improved. Finally, with the increase in the number of sensors in the array, how to develop fault detection and isolation methods with high stability, accuracy, and rapidity needs further research.

## 3. Error Analysis, Modeling, and Calibration

The output data of low-cost consumer MEMS inertial sensors are usually uncalibrated and uncompensated. Due to the limitations of the manufacturing process and manufacturing technology, the sensitive axes of the MEMS inertial sensors produced are not always perfectly aligned, resulting in problems such as shaft misalignment errors and proportional errors [23]. These problems lead to inaccurate output data of inertial devices. Accurate error analysis and modeling of MEMS IMU arrays are the basis of data fusion. Through error analysis, the influence of various error sources on the system is determined, and then the corresponding error model is established, and the effective error compensation is carried out to reduce the impact of the error.

The error of MEMS IMU array is mainly composed of inertial device error and installation error, which includes zero bias, noise, symmetric and asymmetric scale factor error of gyroscope or accelerometer, etc. Installation error is the deviation between the actual installation position of gyroscope or accelerometer and the design position.

Commonly used mathematical methods for error analysis of MEMS inertial devices are mainly divided into the following four types: time series analysis, autocorrelation function method, Power Spectral Density analysis (PSD), and Allan variance analysis [24].

The time series analysis method is the time domain analysis method, and its theoretical core is to assume that a transfer function driven by a white noise source of intensity is able to produce the same statistical and spectral characteristics as the actual black box system. It models a stationary, normally distributed random sequence into a time-dependent sequence driven by white noise occurring at various moments. The autoregressive model (AR) and moving average model (MA) were selected according to the characteristics of spectral three features (crest, trough, and roll drop) of random data of gyroscope angular velocity. ARMA (autoregressive moving average model) is one of three models for error modeling. The advantages of this method are that the error model is simple and recursive, and it can be directly applied to Kalman filter and other filtering algorithms. The disadvantage is that the model is very sensitive and is only suitable for dealing with stable random processes, not for dealing with uncertain power spectrum processes, high-order processes, and wide dynamic range [11,25].

The autocorrelation function method is a classical random data processing method, and it is a form of frequency domain analysis. This method analyzes the characteristics of various random processes in the data according to the autocorrelation characteristics of random data. However, its data acquisition time is long, sometimes even exceeding the instrument life [26].

Power spectrum density analysis is a frequency-domain analysis method. The power spectrum density diagram of random sequence is obtained by Fourier transform. Various random error sources can be identified from the power spectrum density diagram, which is suitable for analyzing periodic or aperiodic signals. However, the analysis results of this method in error modeling of gyroscopes are not easy to understand, and the calculation time and resource consumption are large [11,27].

The Allan analysis of variance is developed on the basis of the power spectral density method. This method utilizes the relationship between the power spectral density function and the Allan variance function of various random errors in the output data of MEMS gyroscope and accelerometer, analyzes and deduces the Allan variance of the output signal, and makes the Allan variance characteristic curve in the log–log coordinate graph to analyze the curve characteristics of random errors in the log–log graph. Allan variance analysis combines time-domain analysis and frequency-domain analysis to form a double-logarithmic random error curve with obvious characteristics, easy to distinguish from each other and simple operation, which can effectively identify various error components and characteristics, so it is widely used in the error analysis of MEMS inertial devices [28].

After the error analysis of the IMU array is completed, the corresponding error model can be established. The following introduces some commonly used error model formulas. The stochastic error model of gyroscope can be written as follows:

(1) $$\begin{array}{l} \varepsilon =\varepsilon_{b}+\varepsilon_{r}+\omega_{g}\\ {\dot{\varepsilon }}_{b}=0\;{\dot{\varepsilon }}_{r}=-\frac{1}{\tau }\varepsilon_{r}+\omega_{r} \end{array}$$

where ε is the overall random drift error of the gyroscope; $\varepsilon_{b}$ is the successive start-up drift, which can be modeled as a random constant, also known as random constant drift; $\varepsilon_{r}$ is the slow-varying drift, which is usually modeled as a first-order Markov process; τ is the correlation time; $\omega_{g}$ is the driving white noise; and $\omega_{r}$ is fast change drift, modeled as a white noise process.

The commonly used error model of gyroscope array can be written as follows, taking the X-axis gyroscope in IMU as an example:

(2) $$\begin{array}{cc} \begin{array}{c} y_{i}=\omega_{i}^{x}+b_{i}+n_{ai}\\ {\dot{b}}_{i}=n_{bi} \end{array} & i=1,2,3,\cdots ,N \end{array}$$

where $y_{i}$ is the measured values of the *i*th gyroscope; $\omega_{i}^{x}$ represents the true X-axis angular rate of the *i*th gyroscope; $b_{i}$ represents the Rate Random Walk (RRW) of the *i*th gyroscope, modeled as the integral of white noise $n_{bi}$; and $n_{ai}$ represents the *i*th gyroscope Angle Random Walk (ARW), modeled as white noise.

The error model of IMU can be written as follows:

(3) $$\left\{\begin{array}{c} \omega =K_{g}N_{g}-\omega_{0}-\delta_{\omega }\\ f=K_{a}N_{a}-f_{0}-\delta_{f} \end{array}\right.$$

where $\omega =\left[\begin{array}{ccc} \omega_{x} & \omega_{y} & \omega_{z} \end{array}\right]$ and $f=\left[\begin{array}{ccc} f_{x} & f_{y} & f_{z} \end{array}\right]$ are the true angular rate and specific force of the gyroscope and accelerometer, respectively; $K_{g}$ and $K_{a}$ are the scale factor and installation relation matrix of the gyroscope and accelerometer, respectively; $N_{g}$ and $N_{a}$ are the measured values of the gyroscope and accelerometer, respectively; $\omega_{0}$ and $f_{0}$ are the zero bias of the gyroscope and accelerometer, respectively; and $\delta_{\omega }$ and $\delta_{f}$ are the noise of the gyroscope and accelerometer, respectively.

The measurements of the *i*th accelerometer triad and *j*th gyroscope triad can then be modeled as follows:

(4) $$\left\{\begin{array}{c} y_{a}^{i}=s+\omega \times (\omega \times r^{i})+\dot{\omega }\times r^{i}+n_{a}^{i}\\ y_{g}^{j}=\omega +n_{\omega }^{j} \end{array}\right.$$

where *s* is the specific force at the origin of the array coordinate system and $r^{i}$ is the location of the *i*th accelerometer triad; ω and $\dot{\omega }$ are the array’s angular velocity and angular acceleration, respectively; and $n_{a}^{i}$ and $n_{\omega }^{j}$ are the measurement errors of the accelerometer triads and gyroscope triads, respectively [29].

After the error analysis and error model of MEMS inertial devices are established, they can be calibrated to determine the parameters of the error model, and then compensate for the deterministic errors in MEMS inertial devices. Calibration is the process of comparing the output of the inertial device with the known reference input to determine a set of parameters that make the output of the inertial device match the reference input. Calibration of the inertial sensors plays an important role in the ultimate accuracy of the IMU array [30].

For the calibration of MEMS IMU array, it is necessary to analyze and model a single inertial device first. On this basis, the error model of the inertial sensor array is established according to the particularity of the array, including not only the calibration factor, installation, coupling, and other errors of the single inertial device, but also the correlation coefficient between the sensors in the array. Calibration of these parameters along with standard parameters are essential to obtain the full capability of an inertial sensor array [29]. The calibration methods of MEMS IMU array can be divided into high-precision rotary table calibration [31], low-precision rotary table calibration and no-rotary table calibration [32]. The most commonly used calibration methods based on the turntable are the speed test and multi-position static test, which usually use the rate test to calibrate gyroscope parameters and the multi-position static test to calibrate accelerometer parameters. The non-turntable calibration method uses the earth gravity acceleration as the external excitation to solve the calibration parameters of the array.

In 2009, Wang of North University of China studied the calibration method of error coefficient of accelerometer array based on angular rate. On the basis of the in-depth study of the inertial measurement combination configuration of the full accelerometer array, he proposed a twelve-accelerometer configuration scheme and carried out the research on the calibration method of the configuration scheme. With the acceleration of earth gravity as the external excitation, the calibration schemes of three different distribution points are calibrated by using a three-axis position rate turntable. The static and dynamic error output models of accelerometers were established, respectively, and the optimal calibration scheme was used to complete the calibration of the scale coefficient of the accelerometer array [33].

**Figure 4:**
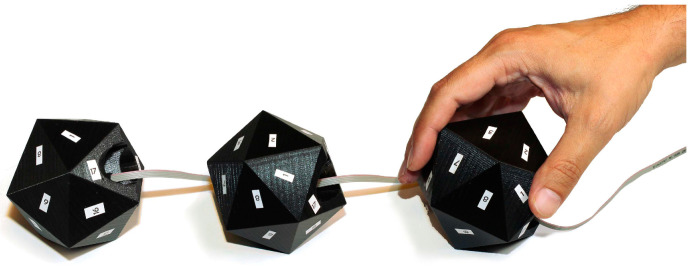
Calibration process of regular 20-hedron to IMU array accelerometer [7].

In 2014, John-Olof Nilsson and others from the KTH Royal Institute of Technology of Sweden designed a positive 20-hedron and established the accelerometer error model of IMU array. The array was placed inside the positive 20-hedron, and static measurement was carried out by sequential transformation of the position of the positive 20-hedron, as shown in Figure 4. It is used to calibrate the constant error of the IMU array accelerometer. After calibration compensation, the measurement accuracy of the IMU array accelerometer is improved by 23 dB [7]. It proves the validity and importance of IMU array calibration and provides an important idea for the later research of the IMU array calibration method.

In 2016, Partick Schopp et al., based in Germany, proposed a self-calibration method for accelerometer array that does not depend on external reference excitation, and only uses the measured values of the accelerometer itself to estimate the accelerometer attitude. In this method, three accelerometers are modeled as a group using an iterative graph optimization algorithm taking the accelerometer attitude and motion as the target variables. The global optimal solution is obtained [34]. It provides a new idea for self-calibration of inertial devices.

**Figure 5:**
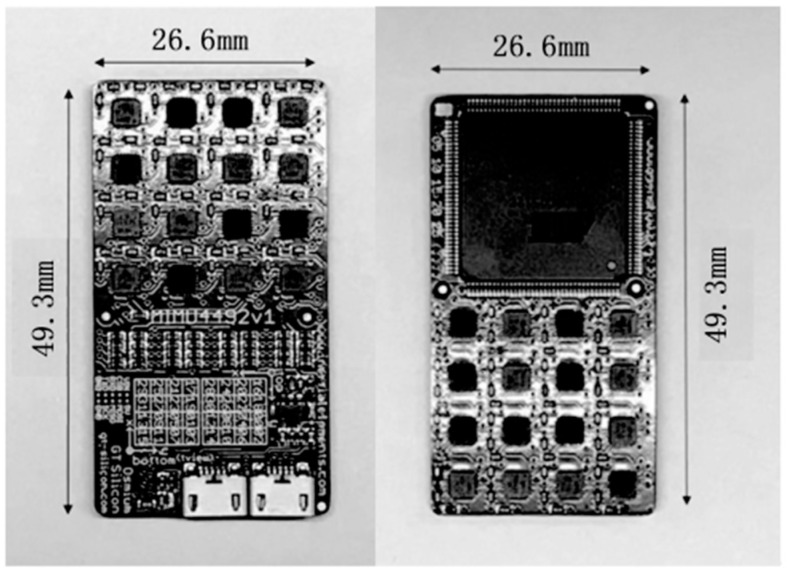
An IMU array containing 32 IMUs [23].

In 2020, Wang Chuang et al. from the University of Shanghai for Science and Technology designed a calibration method based on IMU array. They first established the calibration model of the inertial sensor array, constructed the cost function based on the least square method for its nonlinearity, adopted the L-M algorithm to solve the calibration parameters, and designed the standard positive 20-hedron. To provide enough external excitation for the calibration process, it can also average some random errors and non-modeling errors. They applied the designed calibration method to an IMU array containing 32 MPU-9250 IMUs, as shown in Figure 5. By comparing the calibration results with the technical manual of MPU-9250, the effectiveness of the calibration scheme was verified, and it was applicable to the calibration of both a single IMU and an IMU array [23]. In December of the same year, Zhou et al. from the University of Rocket Force Engineering studied the calibration method of MEMS gyroscope array. They established the error model of the gyroscope array including zero bias error, cross coupling, scale factor, and other parameters, and tested the gyroscope range rate with a high-precision rate turntable. The relationship equation between the theoretical value and the measured value was fitted with the least square method. On this basis, the static weight distribution method was used to calculate the weight assigned after normalization of each gyroscope. Then, the actual output of the calibrated gyroscope array [35] is calculated, and the accuracy of the array is improved by an order of magnitude after error compensation.

In 2021, Hakan Carlsson et al. from Sweden proposed a maximum likelihood estimator for self-calibration of IMU arrays, which can simultaneously estimate calibration parameters and array motion dynamics without the help of external devices and can be used for real-time calibration of small IMU arrays [36]. In the same year, Lukas et al. from the Blocher Group in Germany used a high-precision rotary table to calibrate the constant parameters of the IMU array. After error compensation, the array performed pure inertial navigation based on the initial alignment of the static base, and the position error was only 0.99 m after 30 s. Experiments have proved that an array of 14 MEMS inertial sensors can reduce noise variance by about a factor [37]. In October of the same year, Li Feng et al. from Soochow University established different random noise models, further studied the problem of excessive random noise existing in consumer MEMS inertial devices, analyzed the noise principle and performance, and proposed that when N MEMS inertial sensors are used to form an array, the noise variance can be reduced to [38]. However, as more and more inertial sensors are added to the array, the extent to which the √N rule will remain valid needs to be further investigated.

In 2023, Liu et al. from Wuhan University proposed an online calibration method based on the Levenberg–Marquardt optimization algorithm, which calibrates the deterministic errors of accelerometers and gyroscopes by manually rotating and standing IMU arrays without the help of external reference devices. On-board experiments show that the calibrated IMU array dynamic navigation accuracy is improved by 19.1% on average [39].

In recent years, neural network algorithms have gradually become a research hotspot, and researchers have successively applied machine learning algorithms to array technology. Dong et al. [40] of Xi’an Institute of Microelectronics Technology applied the Long Short-Term Memory (LSTM) neural network algorithm to error correction and compensation of IMU gyroscope array, reducing the bias instability of IMU array by 50% and the Angle random walk by 35%.

**Figure 6:**
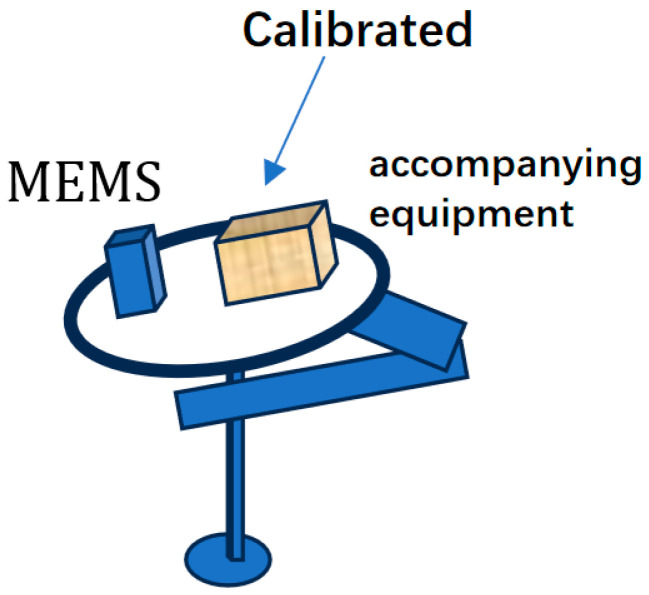
The IMU was calibrated by adjoint testing [38].

In 2024, Wang et al. [41] of Xi’an Institute of Microelectronics proposed an adjoint testing method and weighted fusion method for the calibration and fusion of IMU arrays, respectively. As shown in Figure 6, the adjoint test method obtains error model calibration parameters through a single-chip microcomputer, and calibrates the coordinate system of all sensors in the MEMS array to the same coordinate system, providing a basic guarantee for sensor fusion. Considering the performance difference between sensors, the weighted fusion method adopts a targeted weight allocation scheme, which can improve the error performance index by about ten times. It provides a research idea for low-cost calibration of MEMS IMU array.

## 4. Data Fusion

The method to improve the accuracy of IMU array is to use data fusion to fuse the measurement data of multiple inertial sensors into the output of array. Due to the impact of noise, drift, and error that may exist in a single inertial sensor, data fusion technology fuses multi-dimensional information of the sensor, which can reduce the impact of the error of a single inertial sensor, improve the measurement accuracy of the system, and improve the robustness of the system. Therefore, data fusion technology is the core of MEMS IMU array, and its importance is beyond doubt.

In the early stage of inertial navigation development, researchers proposed a method to improve measurement accuracy through data fusion of redundant information of inertial sensors [42]. For the data fusion of redundant sensors, the simplest method is the least square method, but the least square method uses the measured values of all sensors regardless of good or bad, resulting in the measurement accuracy not being high. Therefore, researchers proposed the weighted least square method [43], which is characterized by the use of weights to ensure that sensors with higher accuracy occupy a greater proportion in data fusion, so as to improve the accuracy of data fusion.

According to the criterion of the least square method, the least squares estimation for the gyroscope angular rate and the specific force of the accelerometer is as follows:

(5) $$\left\{\begin{array}{cc} \begin{array}{c} \hat{\omega }={\left(K_{g}^{-1^{T}}WK_{g}^{-1}\right)}^{-1}K_{g}^{-1^{T}}W{\bar{\omega }}_{i}\\ \hat{f}={\left(K_{a}^{-1^{T}}WK_{a}^{-1}\right)}^{-1}K_{a}^{-1^{T}}W{\bar{f}}_{i} \end{array} & i=1,2,3,\cdots ,N \end{array}\right.$$

where $\hat{\omega }$ and $\hat{f}$ are the least squares estimates of the gyroscope angular rate or the specific force of the accelerometer, respectively. W is a positive definite weighted matrix with appropriate values, and, in particular, when W = I, it is the general least squares criterion. ${\bar{\omega }}_{i}$ is the corrected value of the *i*th gyroscope angular rate measurement and ${\bar{f}}_{i}$ is the corrected value of the *i*th accelerometer specific force measurement.

In addition, the researchers further proposed the recursive least squares method, which can extract the estimated value information from each obtained measurement and be used to modify the estimate obtained in the previous step, so as to make up for the shortcomings of the least squares method which requires the storage of all the measurements.

In 2022, Liang et al. from Xi’an University of Posts and Telecommunication proposed a two-stage fusion noise reduction algorithm based on MEMS array. They performed empirical mode decomposition on gyroscope signals and integrated the Intrinsic Mode Function (IMF) components based on minimum variance and recursive least squares. The fusion angular rate is then superimposed with the mean residual component. The gyroscope Angle random walk processed by this algorithm is reduced by about 68%, and the zero-bias instability is reduced by about 75% [44].

Another commonly used method in sensor data fusion is the weighted average method. The principle of this method is to assign a corresponding weight to the measured value of each sensor and use the size of the weight to reflect the relative reliability of the corresponding sensor. Usually, the sensor with more reliable or higher accuracy is given a higher weight to achieve data fusion. The basic equation of the weighted average method is as follows:

(6) $$\begin{array}{cc} \hat{X}=\frac{\sum_{i=1}^{N}X_{i}\cdot W_{i}}{\sum_{i=1}^{N}W_{i}} & i=1,2,3,\cdots ,N \end{array}$$

where $\hat{X}$ is the result after fusion; $X_{i}$ is the measured value of the *i*th sensor; and $W_{i}$ is the weight corresponding to the *i*th sensor. In this method, weight will directly affect the accuracy and reliability of data fusion, so weight setting is the core of the weighted average method. There are many methods to set the weight, among which the method based on support degree has been studied in the data fusion of the inertial sensor.

In 2007, Yang et al. from the Air Force Engineering University proposed a multi-sensor information fusion algorithm based on support that does not rely on the prior knowledge of sensor observation information and introduced the concept of support by using exponential decay function in information fusion [45]. In 2012, Zhang et al. from Beijing University of Aeronautics and Astronautics applied the support-based information fusion method to MEMS gyroscope arrays. Experiments show that the method can effectively improve the measurement accuracy of the Micro Inertial Measurement Unit (MIMU) [46].

The measurement variance ignored by the above data fusion method based on support is caused by the comprehensive effect of various factors. Later, scholars further proposed an adaptive weighting algorithm, which not only ensures the reliability of the sensor but also minimizes the total variance of the fused target parameters by changing the influence of the variance of the observed values of each sensor on the weighting coefficient [47,48]. Compared with the weighted average method, the estimated value obtained by the adaptive weighted algorithm is closer to the real value.

In addition to the above methods, MEMS IMU array data fusion methods also include Kalman filter [49] and its extension methods, including extended Kalman filter [12] and particle filter [50].

The state equation and measurement equation of the basic Kalman filter are given as follows:

(7) $$\begin{array}{c} \dot{X}(t)\;=\;F(t)X(t)\;+\;G(t)w(t)\\ Y(t)\;=\;H(t)X(t)\;+\;v(t) \end{array}$$

where X(t) is the system state vector; F(t) is the state transition matrix; G(t) is the control input matrix; and w(t) is the control input vector. Y(t) is the system observation vector; H(t) is the observation matrix; and v(t) is the measurement noise.

According to Equations (2) and (7), the equation of state and the equation of measurement are established for gyroscope array.

Let $y=\left[\begin{array}{c} y_{1}\\ y_{2}\\ \vdots \\ y_{N} \end{array}\right]$, $b=\left[\begin{array}{c} b_{1}\\ b_{2}\\ \vdots \\ b_{N} \end{array}\right]$, $n_{a}=\left[\begin{array}{c} n_{a1}\\ n_{a2}\\ \vdots \\ n_{aN} \end{array}\right]$, $n_{b}=\left[\begin{array}{c} n_{b1}\\ n_{b2}\\ \vdots \\ n_{bN} \end{array}\right]$ simply write Equation (2) as

(8) $$\begin{array}{c} y=I\omega +b+n_{a}\\ \dot{b}=n_{b}={\left[\begin{array}{cccc} n_{b1} & n_{b2} & \cdots & n_{bN} \end{array}\right]}^{T} \end{array}$$

When the input angular velocity is zero, the Kalman static filter equation is established, the system state vector $X={\left[\begin{array}{cc} b & \omega \end{array}\right]}^{T}$ is set, the Angle random walk $n_{a}$ is taken as the measurement noise vector, the actual output *y* of the array is taken as the system measurement vector, and the system state equation and measurement equation are established.

(9) $$\left\{\begin{array}{c} \dot{X}(t)={\left[\begin{array}{cccc} n_{b1} & \begin{array}{cc} n_{b2} & \cdots \end{array} & n_{bN} & n_{\omega } \end{array}\right]}^{T}=0\cdot X(t)+I_{N}{\left[\begin{array}{cccc} n_{b1} & n_{b2} & \cdots & n_{bN} \end{array}\right]}^{T}\\ Y(t)=\left[I_{N}\vdots 1_{N}\right]\cdot X(t)+{\left[\begin{array}{cccc} n_{a1} & n_{a2} & \cdots & n_{aN} \end{array}\right]}^{T} \end{array}\right.$$

The static Kalman filter is only applicable to the estimation when the angular velocity input is zero. When the angular velocity input is not zero, the real-time measurement value needs to be fused with the previous state estimation to realize the dynamic update of the system state estimation. The Kalman dynamic filter equation is established, the system state vector $X={\left[\begin{array}{cccc} b_{1} & b_{2} & \cdots & b_{N} \end{array}\right]}^{T}$ is set, and the actual output vector of the gyroscope array is processed differently to eliminate the influence of the unknown real angular velocity on the measured value.

(10) $$\left\{\begin{array}{c} \dot{X}(t)={\left[\begin{array}{cccc} n_{b1} & n_{b2} & \cdots & n_{bN} \end{array}\right]}^{T}=0\cdot X(t)+I_{N}{\left[\begin{array}{cccc} n_{b1} & n_{b2} & \cdots & n_{bN} \end{array}\right]}^{T}\\ \left[\begin{array}{c} Z_{1}(t)\\ Z_{2}(t)\\ \vdots \\ Z_{N}(t) \end{array}\right]=\left[\begin{array}{c} Y_{2}(t)-Y_{1}(t)\\ Y_{3}(t)-Y_{2}(t)\\ \vdots \\ Y_{1}(t)-Y_{N}(t) \end{array}\right]=\left[\begin{array}{cccc} -1 & 1 & \cdots & 0\\ 0 & -1 & \cdots & 0\\ \vdots & \vdots & \vdots & \vdots \\ 0 & 0 & \cdots & -1 \end{array}\right]\cdot X(t)+\left[\begin{array}{c} n_{a2}-n_{a1}\\ n_{a3}-n_{a2}\\ \vdots \\ n_{a1}-n_{aN} \end{array}\right] \end{array}\right.$$

According to the above state equation and measurement equation, a Kalman filter is constructed to obtain the optimal estimate of the random walk of the state rate, and then the optimal estimate $\hat{\omega }$ of the input angular rate is extracted from it.

In 2016, Liu et al. from Rocket Army Engineering University proposed a signal fusion method of MEMS gyroscope array based on an optimized Kalman filter. Based on the linear measurement of the MEMS gyroscope array signal, a Kalman filter is constructed and the steady-state value of the gain matrix of the Kalman filter is used to estimate the angular rate. The calculation of gain and mean square error in each step is avoided, and the process of optimal estimation is improved. The optimized Kalman filter reduces the complexity and computation of the processing. Finally, the fusion estimation of diagonal velocity is realized. Under this method, the noise of the array composed of six gyros is reduced by 144.2 times under static condition and 18.18 times and 5.36 times under constant rate and sinusoidal rate, respectively [51].

Since the Kalman filter is only applicable to linear systems, when the system model is nonlinear, a model approximation error will be caused. In the IMU array, the gyroscope and accelerometer can provide angular velocity measurement information at the same time, and the output of acceleration and angular velocity are nonlinear, thus the extended Kalman filter is derived. The nonlinear problem is approximately transformed into a linear problem by discarding higher order terms above the second order through Taylor expansion [52].

In 2018, Xing Li from the Nanjing University of Aeronautics and Aeronautics proposed an angular velocity estimation method of IMU array based on an improved extended Kalman filter, established the state equation and measurement equation of angular velocity fusion estimation of IMU array, analyzed and deduced the improved extended Kalman filter equation when state noise and measurement noise were correlated, and realized the fusion estimation of angular velocity information in the array [53].

For the data fusion of MEMS inertial sensor array, based on the above basic methods, the researchers also combined and extended it, and put forward many new data fusion methods.

In 2020, Hiroyuki Kamata et al. [54] from Japan proposed a signal processing filtering method for MEMS gyroscope array that is easy to implement on FPGA. They established an interference noise model and abnormal noise model, and restored gyroscope performance by suppressing interference noise and dynamically removing outlier noise, including angular random walk and bias instability. For MEMS gyroscope sensors with mutual interference and poor performance, the array containing 32 consumer MEMS IMUs is raised to nearly the ideal gain value $1/\sqrt{32}$ under the effect of this filtering method. In the same year, Liang et al., Xi’an University of Posts and Telecommunications, proposed a Wavelet Compressive Fusion (WCF) wavelet efficient optimal estimation algorithm for MEMS arrays. The algorithm uses the compression characteristic of multi-scale wavelet transform to compress the original signal output of MEMS inertial sensor array based on support fusion, and then performs threshold processing on the fused wavelet coefficients. This method increases gyroscope zero bias instability, Angle random walk, and rate slope by 8.0, 8.0, and 9.5 dB, respectively [55].

In 2023, Miao et al. of the Beijing Institute of Technology [56] proposed a gyroscope array fusion algorithm based on the combination of neural network and Kalman filter. LSTM neural network was used to calculate the confidence of gyroscope, and multi-layer feedforward network Back Propagation (BP) was used to identify gyroscope faults and reduce the measurement data utilization of the faulty gyroscope. The method can reduce the mean absolute error of gyroscope array by 80.25% and the root mean square error by 81.39% in the case of faulty gyroscope array. In the same year, Wei et al. from the Shanghai University of Engineering Technology designed an array IMU hardware platform based on redundant measurement information, and improved the extended Kalman filter fusion algorithm by using the iterative reweighted least square method, which realized that the random error of array IMU was reduced by three to five times compared with that of a single IMU [57].

## 5. Fault Detection and Isolation

MEMS IMU array consists of multiple inertial sensors, including a large number of accelerometers and gyroscopes [22]. The failure of individual sensors in a large number of sensors is inevitable, that is, the sensor is in an abnormal working state. Once the sensor fails, its output measurement data will become unreliable, leading to a lot of uncertainty in the entire system. Sun et al., Nanjing University of Aeronautics and Astronautics, divided sensor faults of navigation systems into the following three types: inaccurate output, unreasonable output, and inconsistent output [58].

Fault Detection and Isolation technology can detect and identify the faults of a single or multiple sensors in the inertial sensor array, and take timely measures to isolate the faulty sensors, thereby improving the stability and reliability of the entire system [59]. FDI approaches can be divided into the following categories: hardware-based approach, analyzing-based approach, and AI-based approach.

In 2014, Drew E. Bittner et al. developed a Fault Detection, Isolation, and Recovery (FDIR) architecture for large-scale IMU arrays. Used to identify anomalies and error data output from a large number of real-time parallel data, the architecture uses K-nearest Neighbors (KNN) to calculate the difference between all sensor measurements in unit time several times, and thus determine the corresponding tolerance N and establish a neighboring value K. When the difference between the measured value of a sensor and at least K sensors is within the tolerance value N, the measured value of the sensor is considered to be “good”, otherwise it is considered to be “bad”. The abnormal IMU is identified by the above method, and the wrong output is prevented from being included in the state estimation. Taking 16 IMUs as examples, they calculated the difference for a second time and set the adjacent value to 4. The Monte Carlo simulation method was used to test the reliability of the FDIR architecture under various random faults, and the results showed that the architecture could process a large amount of IMU measurement information and had good robustness [60].

At present, there is little research on fault detection and isolation of MEMS IMU arrays, but the array technology of sensors is a form of redundancy technology. Therefore, the best solution to improve the fault detection and isolation performance of MEMS IMU array sensors is to implement redundant sensor configuration, which can not only increase the fault tolerance rate of the system, but also improve the measurement accuracy of the system [61]. The inertial navigation system can be divided into system level redundancy and device level redundancy according to the redundancy level. The device level redundancy is to configure more sensors on the basis of the traditional structure of three axes and six tables.

For the fault detection of Redundant Inertial Measurement Unit (RIMU), common methods include the direct comparison method based on equivalent space principle [62], Generalized Likelihood Ratio (GLR) [63], Optimal Parity Test (OPT) [64], and Singular Value Decomposition (SVD) [65].

The direct comparison method is applicable to the hard fault detection of inertial devices. This method takes advantage of the principle that there must be linear correlation between any four vectors, $X_{1}$, $X_{2}$, $X_{3}$ and $X_{4}$, in three-dimensional space. The equation is as follows:

(11) $$aX_{1}+bX_{2}+cX_{3}+dX_{4}=0$$

where *a*, *b*, *c*, and *d* are real constants that are not all zero. Therefore, in a redundant IMU, the measured values of the gyroscope (accelerometer) along any four directions also have this linear correlation. Taking a gyroscope with regular dodecahedral redundancy as an example, the measurement error is ignored, which can be obtained by the above Equation (11).

(12) $$(m_{1}-m_{2})\cos\alpha -(m_{3}+m_{4})\sin\alpha =0$$

Equation (12) shows that if the measured values of the four gyroscopes meet the above equation, it means that the four gyroscopes are not faulty, otherwise it means that at least one of the four gyroscopes is faulty. Through the above steps, the fault detection problem can be transformed into a logical judgment problem of linear correlation equations. This method of detecting faults by comparing the measured values of each gyroscope is also called the parity detection method, and the linear correlation equation of Equation (12) is also called the parity equation. Similarly, by combining multiple gyroscopes, we can set up odd equations, identify all the equations whose results are not 0 as 1, and then establish the truth table of fault isolation, through which fault detection and fault gyroscopes can be quickly isolated. In particular, when three or more gyros fail, this method can only be used for fault detection and cannot be used for fault isolation.

The generalized likelihood ratio method is applicable to the soft fault detection of inertial devices. In this method, the concept of parity vector is introduced. When a sensor fails, the corresponding fault vector will appear, resulting in the inconsistency between the parity vector with fault and that without fault, which provides the basis for fault detection. The fault detection decision function is established according to the statistical characteristics of the parity vector ρ in the fault-free hypothesis $H_{0}$ and the fault-free hypothesis $H_{1}$, and the fault decision criterion is established through the pre-set threshold. When a sensor is determined to have a fault, the likelihood function of the parity vector ρ is further analyzed to determine the specific fault sensor and isolate it.

The optimal parity vector method is an advanced method of the parity vector method. It not only uses the parity of the output value of the sensor to detect faults, but also introduces an optimization algorithm to improve the accuracy and efficiency of fault detection.

Singular value decomposition method uses the principle of matrix singular value decomposition to construct the output matrix from the output data of the inertial device and performs singular value decomposition to obtain the singular value and corresponding singular vector. Abnormal or faulty sensors usually cause some singular values to deviate significantly from the normal singular value distribution. When the singular vector of a sensor deviates significantly from other singular vectors, it indicates that the sensor has a fault or anomaly.

In addition to the above methods, in recent years, many wavelet packet decomposition techniques, such as fuzzy decision making, support vector machine (SVM), and wavelet packet decomposition, have been proposed in the literature. Methods such as WPD and principal component analysis (PCA) are introduced into the fault diagnosis of redundant IMUs.

In 2008, Li et al. from the Air Force Engineering University used multiple gyroscopes to measure the same variable repeatedly, used fuzzy decision to evaluate the quality of odd-even residuals, and designed an adaptive gradient fault tolerance method of redundant IMU. This method can play a good role in detecting and tolerating the graded faults of up to two gyroscopes [66].

In 2015, Li Yong from the Nanjing University of Aeronautics and Astronautics used wavelet packet decomposition to extract the energy features of gyroscopes, combined with support vector machine classifiers to carry out gyroscope fault diagnosis, and built a gyroscope fault diagnosis system based on a fuzzy support vector machine and incremental learning algorithm [67].

In 2020, Hao et al. proposed an improved principal component analysis (PCA) fault detection algorithm based on odd-even space generation to make up for the shortcomings of PCA for dynamic fault detection of redundant IMUs. This method uses odd-even vectors to isolate dynamic variables to eliminate the influence of dynamic variables on fault detection, uses the principal component analysis method to detect sensor information in real-time, and transposes the original data set to the feature plane to form patterns, thus achieving accurate separation of normal and fault modes of IMU [68].

For MEMS IMU arrays, the fault detection and isolation methods can be further studied by referring to the above outlined IMU fault detection and isolation methods.

## 6. Summary and Prospect

At present, the research on MEMS IMU array technology mainly has the following characteristics:

(1) The research focuses on calibration methods. Most calibration methods are based on high-precision turntables, and a few researchers have proposed self-calibration methods without the help of external equipment.
(2) In the research of data fusion methods, with Kalman filter and its extension method as the main, the stability of array measurement stability is improved by data fusion.

To sum up, the following aspects need further study:

(1) To study low-cost calibration methods, new self-calibration methods can be considered to reduce calibration costs, and how to accurately calibrate inertial sensor arrays with different installation error angles and large installation error angles.
(2) As data fusion technology is the core of MEMS IMU array technology and is directly related to the accuracy of the array, how to further improve the fusion accuracy and reliability of MEMS IMU array needs to include optimizing and improving the existing methods. Further research can be conducted on the data fusion method combining the Kalman filter and its extension method with neural network.
(3) When the number of sensors in the inertial sensor array becomes larger and larger, the extent to which the rule will maintain its effectiveness remains to be further studied.
(4) With the increase in the number of inertial sensors in MEMS IMU array, the collected information is complex and the data are large, so it is very important to study fault detection and isolation methods with high stability, accuracy, and rapidity.

Using low-cost, low-precision MEMS inertial devices to compose inertial sensor array, through data fusion, error analysis, modeling and calibration, fault detection and isolation, and other technologies, can greatly reduce its random error and improve the accuracy, so that the use of low-cost, low-precision inertial devices to compose high-precision inertial navigation systems is possible. This paper summarizes some of the literature in the field of inertial sensor array in recent years, discusses the research status and key technologies of MEMS inertial sensor array technology, and puts forward some thoughts on the future research direction.

## Data Availability

Not applicable.
